# Mixed Effect Modeling of Dose and Linear Energy Transfer Correlations With Brain Image Changes After Intensity Modulated Proton Therapy for Skull Base Head and Neck Cancer

**DOI:** 10.1016/j.ijrobp.2021.06.016

**Published:** 2021-06-19

**Authors:** Grete May Engeseth, Renjie He, Dragan Mirkovic, Pablo Yepes, Abdallah Sherif Radwan Mohamed, Sonja Stieb, Clifton Dave Fuller, Richard Wu, Xiadong Zhang, Liv Bolstad Hysing, Helge Egil Seime Pettersen, Camilla Hanquist Stokkevåg, Radhe Mohan, Steven Jay Frank, Gary Brandon Gunn

**Affiliations:** * The University of Texas MD Anderson Cancer Center, Department of Radiation Oncology, Houston, Texas;; † Haukeland University Hospital, Department of Oncology and Medical Physics, Bergen, Norway;; ‡ The University of Bergen, Department of Clinical Science, Bergen, Norway;; § The University of Texas MD Anderson Cancer Center, Department of Radiation Physics, Houston, Texas;; ║ Rice University, Physics and Astronomy Department, Houston, Texas;; # The University of Bergen, Department of Physics and Technology, Bergen, Norway

## Abstract

**Purpose:**

Intensity modulated proton therapy (IMPT) could yield high linear energy transfer (LET) in critical structures and increased biological effect. For head and neck cancers at the skull base this could potentially result in radiation-associated brain image change (RAIC). The purpose of the current study was to investigate voxel-wise dose and LET correlations with RAIC after IMPT.

**Methods and Materials:**

For 15 patients with RAIC after IMPT, contrast enhancement observed on T1-weighted magnetic resonance imaging was contoured and coregistered to the planning computed tomography. Monte Carlo calculated dose and dose-averaged LET (LET_d_) distributions were extracted at voxel level and associations with RAIC were modelled using uni- and multivariate mixed effect logistic regression. Model performance was evaluated using the area under the receiver operating characteristic curve and precision-recall curve.

**Results:**

An overall statistically significant RAIC association with dose and LET_d_ was found in both the uni- and multivariate analysis. Patient heterogeneity was considerable, with standard deviation of the random effects of 1.81 (1.30–2.72) for dose and 2.68 (1.93–4.93) for LET_d_, respectively. Area under the receiver operating characteristic curve was 0.93 and 0.95 for the univariate dose-response model and multivariate model, respectively. Analysis of the LET_d_ effect demonstrated increased risk of RAIC with increasing LET_d_ for the majority of patients. Estimated probability of RAIC with LET_d_ = 1 keV/*μ*m was 4% (95% confidence interval, 0%, 0.44%) and 29% (95% confidence interval, 0.01%, 0.92%) for 60 and 70 Gy, respectively. The TD_15_ were estimated to be 63.6 and 50.1 Gy with LET_d_ equal to 2 and 5 keV/*μ*m, respectively.

**Conclusions:**

Our results suggest that the LET_d_ effect could be of clinical significance for some patients; LET_d_ assessment in clinical treatment plans should therefore be taken into consideration.

## Introduction

The main rationale for using intensity modulated proton therapy (IMPT) in the treatment of head and neck cancers (HNC) is the ability to create highly conformal treatment plans with reduced normal tissue doses and potentially lower complication rates compared with photon therapy.^1^ Protons are considered to be more biologically effective than photons. In proton treatment planning and delivery this is accounted for by using a fixed value of 1.1 for the proton relative biological effectiveness (RBE).^2^ However, the RBE of protons is not constant; it varies depending on a complex combination of dose, clinical endpoint, tissue *α*/*β*, and the linear energy transfer (LET).^3–5^ An approximately linear increase in RBE with increasing LET (keV/*μ*m) has been shown for dose- and energy ranges relevant for clinical use.^5^ As LET increases with increasing depth, its maximum is at the end of the proton range, typically close to the clinical target volume (CTV) border. Further, IMPT treatment planning studies have reported elevated LET and increased biological dose in organs at risk (OAR) in close proximity to the CTV.^6^ Questions are therefore raised whether increased RBE in OAR adjacent to the CTV could lead to radiation-associated normal tissue injury with subsequent development of adverse effect.

The clinical evidence of a causal effect of LET with radiation-associated side effects is limited and inconclusive. Based on voxel-level analysis of posttreatment imaging data, a few studies have reported correlations between LET and regions of radiation associated brain image change (RAIC) in pediatric and adult patients treated with proton therapy.^7–9^ In contrast, no such correlation was found in a recent study including 50 patients treated with passive scattering proton therapy (PSPT), where several different methods were used to investigate LET and RAIC associations.^10^

As HNC near the skull base often consists of complex target volumes surrounded by dose-limiting critical organs, highly modulated proton beams with steep dose gradients are required to create optimal treatment plans. This may potentially lead to high LET and increased biological effect in critical structures compared with what is indicated by the fixed RBE weighted dose distribution.^6,11^ Moreover, our group recently characterized a cohort of patients with skull base HNC with RAIC events after treatment with proton therapy.^12^ These lesions were overlapping or located just outside the CTV border, indicating a potential increased biological effectiveness of protons due to elevated LET in the dose fall-off area. Therefore, the purpose of the current study was to explore dose and LET correlations with RAIC in a subgroup of patients treated with IMPT for skull base HNC.

## Methods and Materials

### Patients and treatment

The study cohort included 15 patients with HNC at the skull base who had been diagnosed with RAIC after IMPT. These 15 patients were identified after review of available magnetic resonance (MR) reports and images for development of RAIC in 85 patients previously treated with IMPT at our center between December 2010 and June 2018. All patients were participants in 1 of 2 prospective clinical trials (ClinicalTrials.org identifiers: NCT 00991094 and NCT 01627093) and had provided study-specific written informed consent.

The patients’ treatment plans had been generated in the Eclipse Treatment planning system (Varian Medical Systems, Palo Alto, CA). Treatment planning was based on non-contrast CT images acquired with the patient in supine position and immobilized with a posterior customized mold and thermoplastic mask. CTV definitions had been manually performed and peer-reviewed before treatment planning. The typical beam arrangements consisted of multiple beams with large angular separation to spread out the placement of potential high-LET and with the majority of patients being treated with 1 posterior and 2 left and right anterior oblique beams (Fig. E1). Each treatment plan used a simultaneous integrated boost technique and was individually optimized to obtain optimal CTV coverage while minimizing dose to surrounding normal tissues.

### RAIC definition, image registration, and Monte Carlo simulations

RAICs had initially been assessed on posttreatment surveillance MRIs, which were routinely acquired every 3 to 4 months during the first 2 years after treatment completion, then every 6 months until 5 years, and annually thereafter. The MRI findings defined as RAIC included gadolinium contrast-enhanced brain lesions on T1-weighted (T1_w_) sequences, accompanied by increased signal intensity/edema and/or cysts on T2-weighted (T2_w_) sequences.^13^ Retrospectively, a second review with verification of the RAIC diagnosis was performed by 2 board-certified radiation oncologists (GBG and SJF). Both the radiologists and the oncologists were blinded to the dose and LET distributions and were not involved in the further statistical analysis and modeling of the dose and LET correlations with RAIC. In a typical RAIC evolution, the initial phase is often followed by progression of the lesion.^14^ The majority of the patients had several consecutive MRIs after RAIC diagnosis with lesions of varying (increasing/decreasing) size; for the current analysis we considered the contrast enhanced lesions from the earliest T1_w_ MRI with observed RAIC to be the most appropriate surrogate for the origin of the radiation associated injury. The earliest MRI with RAIC and the treatment planning CTs were automatically registered (rigid), and the result of the image registration was evaluated by visual inspection and manually modified if deemed necessary. Figure E1 shows an example of a contrast enhanced lesion visible on the T1_w_ MRI sequence and the contoured lesion propagated on the treatment planning CT with the 40 to 70 Gy(RBE) isodose lines overlaid.

For characterization of the proton beam quality either the full LET spectrum or an average LET at each point could be used. The LET average is typically calculated using either the arithmetic mean of the LET fluence spectrum (track averaged [LET_t_]) or by weighting the LET by the dose it deposits in each point(dose averaged LET [LET_d_]).^15^ For therapeutic proton beams, LET_d_ is considered to be more appropriate than LET_t_.^5,16^ To obtain accurate dose and LET distributions for the brain tissue and the RAIC lesions, the treatment plans were recalculated using an in-house developed Monte Carlo system: the Fast Dose Calculator (FDC). The FDC is a track-repeating algorithm for proton therapy, validated for scanning beams.^17–19^ The FDC algorithm calculates the dose and unrestricted LET_d_ based on the patient’s treatment plan and the assigned planning CT. The LET_d_ includes primary and secondary protons and is computed using a step-by-step approach previously described by Cortes-Giraldo and Carabe,^20^ where LET_d_ is calculated from pregenerated tables of stopping power obtained from GEANT4.^21^ The resulting Monte Carlo doses and LET_d_ distributions were extracted at the voxel level for each patient, whereupon each voxel within a contoured lesion was defined as one single RAIC event (ie, binary response value = 1), with the voxels outside the lesions (in the brain tissue) defined as nonevents (ie, binary response value = 0).

### Modeling and risk estimation

The data material consisted of multiple voxels from each patient, including the voxel-wise associated dose, LET_d_, and binary response values. Because the data were clustered within patients, mixed effect logistic regression was used to investigate the association between RAIC, dose, and LET_d_.^22–24^ In contrast to a standard logistic regression model, mixed effect logistic regression takes into account patient heterogeneity and the within-patient correlation of the data. It allows for variation of the model intercept and/or predictor coefficients and provides estimates of the effects that are constant across the patients (fixed effects) as well as the effects that vary across patients (random effects). The main predictors in the current models were the physical dose and the LET_d_, and we assumed that the effect of these predictors varied between the patients. Therefore, the univariate and multivariate analyses were performed with estimation of the fixed and random effects of both dose and LET_d_. In addition to dose and LET_d_ we included an interaction term (LET_d_:dose) in the multivariate model. Interaction terms can be applied during modeling to investigate whether a predictor has a different effect on the outcome depending on the value of another predictor. Because the scale of LET_d_ and dose differ, Z-standardization of the predictor variables was performed before modeling (mean = 0, standard deviation = 1).

Model fits were evaluated using the Akaike information criterion (AIC), log likelihood, pseudo R^2^, and Brier score. The models’ ability to discriminate between voxels with and without RAIC was evaluated using the receiver operating characteristic (ROC) curve and the calculated C-index (area under the curve [AUROC]). As an additional discriminative measure, we generated precision-recall (PR) curves, as they are an appropriate and useful supplement to ROC curves for evaluating performance in imbalanced data sets with rare events and where the minority class is of interest, as with the current study.^25^ Both ROC curves and PR curves are model-wide evaluations; for a range of different probability thresholds, the ROC curves plot the trade-offs between the true positive rate versus the false positive rate, whereas the PR curves plot the precisian versus the recall.

Cluster bootstrapping was used for internal model validation. The cluster bootstrapping procedure involves resampling of patients with replacement, rather than resampling of individual observations. This resampling strategy has been proven superior over both resampling of individual observations and a 2-level successive resampling of patients and observations.^26^ For the current analysis this implied that patients (including all the voxels from each of the selected patients) were resampled with replacement (number of samples = 1000), whereupon the model was fit on each of the bootstrap samples and performance measures extracted. The modeling was performed in R, version 3.6.0^27^, using the *glmer* function from the *lme4* library.^28^

## Results

**Fig. 1.**
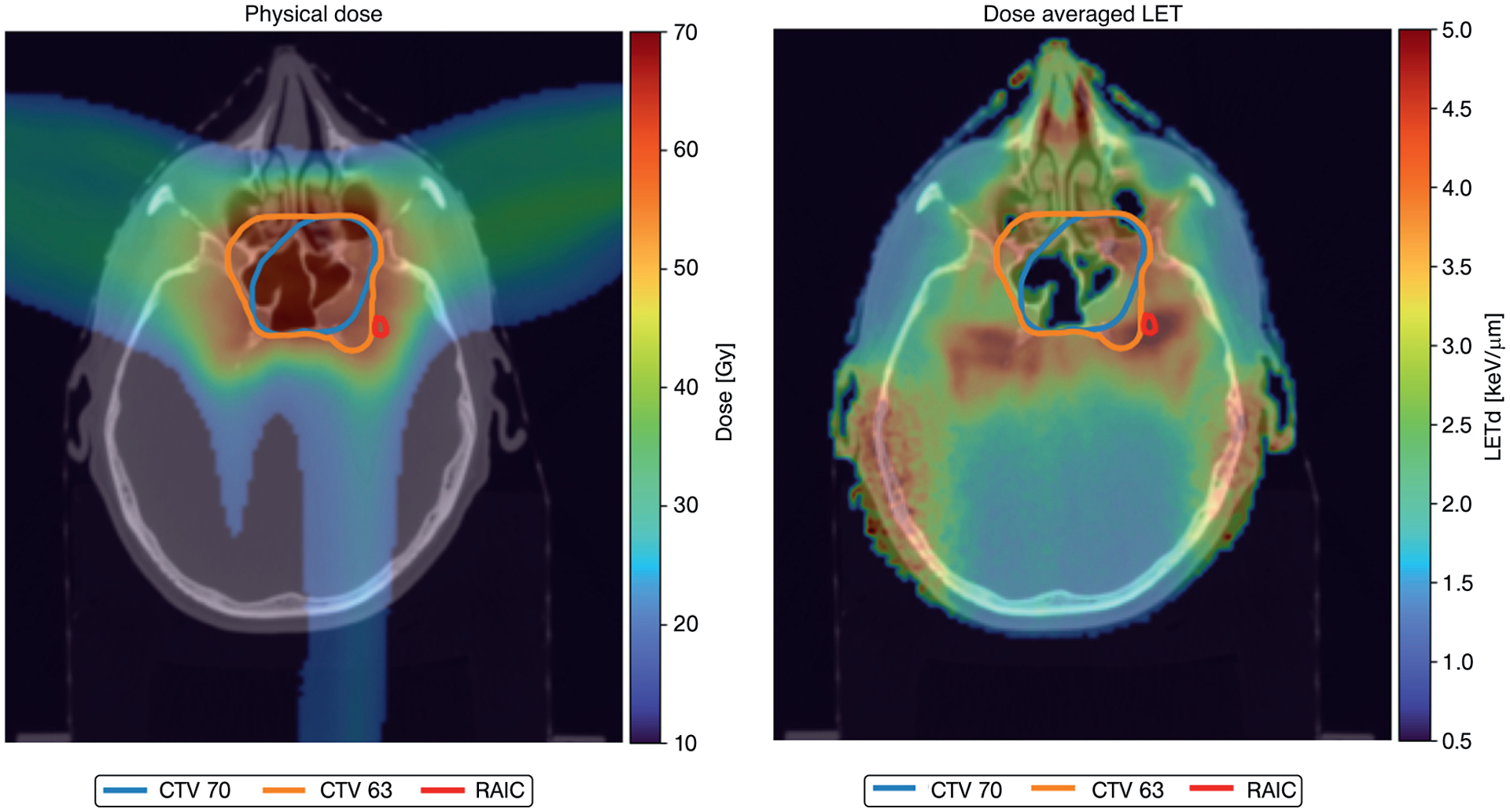
Example of dose distribution (physical dose) to the left and the dose-averaged linear energy transfer (LET_d_) distribution to the right. The high LET_d_ in low-dose regions laterally are due to secondary radiation. Radiation-associated brain image change (RAIC) (red contour): the contoured contrast enhanced lesion from the T1 weighted magnetic resonance imaging (MRI).

The patient characteristics are presented in Table 1. All patients had skull base and/or intracranial involvement with typical disease extent to sphenoid sinus, cavernous sinus, and dura. Thirteen (86.7%) lesions were in the temporal lobe(s) and 2 (13.3%) in the frontal lobe. The median (range) lesion volume was 0.2 cm^3^ (0.1–1.1 cm^3^). Ten of the patients had lesion volumes less than 0.3 cm^3^. The number of voxels in the lesions ranged between 195 and 5365, whereas the number of voxels in the irradiated brain area ranged between 6157 and 49,238. The proportion of voxels with RAIC in the total data set was 6%. The median (range) LET_mean_ and D_mean_ (RBE = 1.1) in the lesions were 3.61 keV/*μ*m (2.82–5.59 keV/*μ*m) and 63.5 Gy(RBE) (42.2–69.0 Gy[RBE]), respectively. The highest LET_d_ value in a lesion was 8.04 keV/*μ*m and the highest LET_d_ value in the brain tissue was 10.69 keV/*μ*m. An example of dose and LET_d_ distribution including RAIC and CTV contours is displayed in Figure 1.

**Fig. 2.**
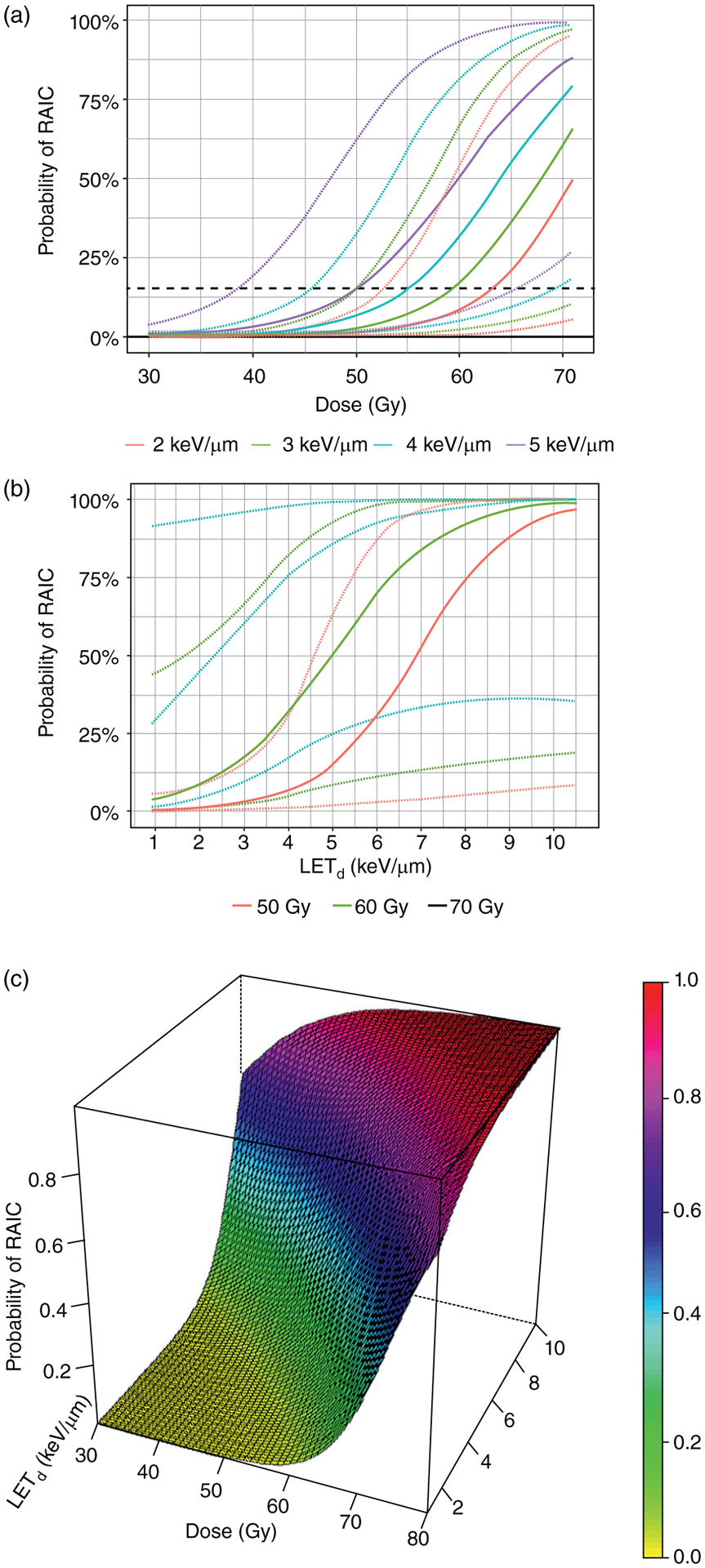
Probability curves (solid lines) including 95% confidence intervals (dotted lines) for (a) 4 different dose-averaged linear energy transfer (LET_d_) values in and for (b) 3 different dose levels in. Dashed horizontal lines correspond to (a) 15% and (b) 5% probability of radiation associated brain image change (RAIC). (c) The corresponding surface plot is displayed. All the plots were generated from the multivariate model.

The fixed effects represent the overall (constant) effect of the predictors on RAIC. There was a positive and statistically significant correlation between RAIC and dose, as well as between RAIC and LET_d_ in both the univariate and multivariate models (Table 2). We further found a small but significant interaction between LET_d_ and dose; that is, as dose increases, the effect of LET_d_ decreases and vice versa. As shown by the negative coefficient sign, the combined effect of dose and LET_d_ was therefore less than the sum of the individual effects. The conditional effects of LET_d_ and dose are illustrated in Figure E2. Based on the multivariate model we generated probability curves for several LET_d_ values and dose levels (Fig. 2a,b). The corresponding surface plot of the model is displayed in Figure 2c. The TD_15_ (the dose for 15% probability of RAIC) were estimated to be 63.6 and 50.1 Gy with LET_d_ equal to 2 and 5 keV/*μ*m, respectively (Fig. 2a). A rapid increase in RAIC risk could be observed when doses exceeded 60 Gy even for lower LET_d_ values; for LET equal to 1 keV/*μ*m the estimated risk of RAIC was 4% (95% confidence interval [CI], 0%−0.44%) at 60 Gy versus 29% (95% CI, 0.01%−0.92%) at 70 Gy (Fig. 2b).

**Fig. 3.**
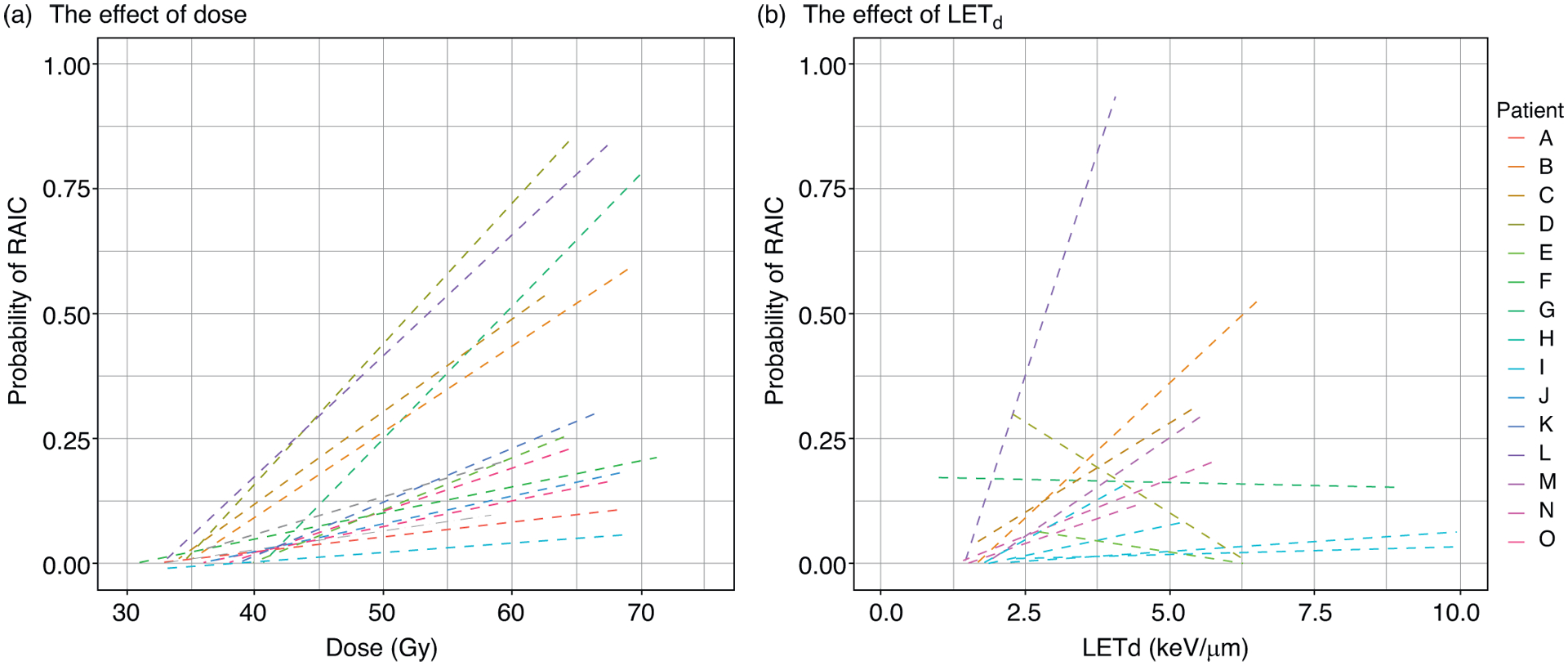
(a) Dose and (b) dose-averaged linear energy transfer (LET_d_) plotted versus estimated risk of radiation associated brain image change (RAIC). Individual trend lines are generated for all values of LET_d_ and dose using a glm smoothing function (y~x).

The random effects are associated with patient heterogeneity. The intraclass correlation (ICC) was 0.77. The standard deviations (95% CI) of the random effects were 1.81 (1.30–2.72) for dose and 2.68 (1.93–4.93) for LET_d_. The interpatient variation is illustrated in Figure 3, where the risk estimates are plotted as a function of dose and LET_d_ and with individual trend lines generated for each of the patients. A distinct difference between dose and LET_d_ could be observed; although the effect of the dose was moderate for some patients, there was still a clear trend of increasing risk with increasing dose (Fig. 3a). For LET_d_, on the other hand, the trend was less consistent, with a positive LET_d_ effect for the majority of the patients; however, with a negative LET_d_ effect for 3 of the patients (Fig. 3b). Besides an overall lower LET_d_ in the RAIC regions compared with the rest of the brain tissue in these 3 patients, our analysis revealed nothing specific regarding number of beams (2–3), beam arrangements, dose distribution, CTV location, or disease extent that could explain this finding. The interpatient variation resulted in large uncertainties in RAIC predictions. The probability curves with 95% prediction interval are displayed in Figure E3.

**Fig. 4.**
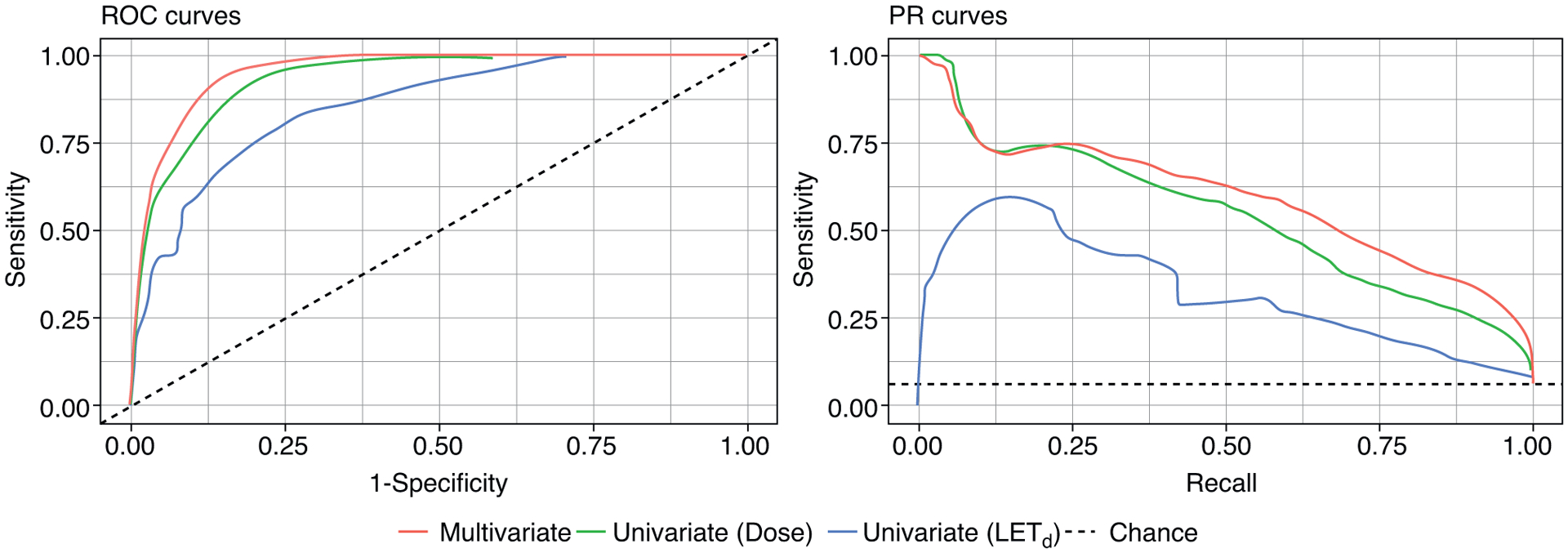
Receiver operating characteristic (ROC) curves (left) and precision-recall (PR) curves (right). The dashed lines represent a no-skill model (area under the receiver operating characteristic [AUROC] = 0.5 and area under the precision recall [AUPR] = 0.06).

Model fit and performance measures are displayed in Table 2. The AUROC and the area under the precision recall curve (AUPRC) were 0.85 and 0.33 for the univariate model with LET_d_ as predictor, and 0.93 and 0.54 for the univariate model with dose as predictor, respectively. The performance of the multivariate model was slightly improved with an AUROC and AUPRC of 0.95 and 0.59, respectively (Fig. 4). Cluster bootstrapping was used for internal validation of the multivariate model. The mean AUROC from the cluster bootstrap procedure was 0.95 (95% CI, 0.92–0.97), whereas the mean AUPRC was 0.58 (95% CI, 0.41–0.71).

We further performed a subgroup analysis to investigate dose and LET_d_ correlations with RAIC when all voxels with doses below 40 Gy were removed from the data set. The results from this analysis were consistent with the main analysis, with significant associations with dose and LET_d_ in both the uni- and multivariate analysis, and a significant interaction between LET_d_ and dose. Compared with the main model, the AUROCs were slightly reduced to 0.84 for both the univariate models and to 0.90 for the multivariate model. The parameter estimates from the analysis are displayed in Table E1 with ROC and PR curves in Figure E4.

## Discussion

In the current study, dose and LET_d_ associations with RAIC in patients treated with IMPT for HNC at the skull base were explored using voxel-level data and mixed effect logistic regression modeling. Our result demonstrated positive and significant dose and LET_d_ associations with RAIC in all models and a slightly improved ability to discriminate between voxels with and without RAIC when LET_d_ was included as predictor. We further found that the effect of dose and LET_d_ varied considerably between patients, resulting in wide CIs and large uncertainties in predictions.

A few previous studies have aimed to investigate the associations between elevated LET and regions with RAIC by analyzing voxel level data. In 34 pediatric patients treated with PSPT for ependymoma, Peeler et al^9^ reported a significant correlation between hyper-intensities on T2-Fluid-attenuated inversion recovery images and LET_t_. They developed a model with LET_t_ and dose as predictors and showed that the estimated tolerance dose for 50% risk (TD_50_) of image change in a voxel was reduced when LET_t_ increased. Similar findings were reported by Eulitz et al,^8,29^ investigating correlations between LET_t_ and contrast enhanced lesions from T1_w_ MRIs in adult patients treated with PSPT for glioma. They found improved predictive performance when including LET_t_ in the dose-response models; and, as in Peeler et al, a reduction in TD_50_ was observed with increasing LET_t_. Bahn et al^7^ developed a model for patient-specific predictions of the local risk of image change based on the treatment plan using voxel level data from a large cohort (n = 110) of low-grade gliomas treated with pencil beam scanning. They showed that the location of RAIC mainly occurred in regions with combined high dose and LET_d_ and not at random. In all these studies, the LET-RAIC associations were analyzed using generalized linear models, assuming uncorrelated observations. Similar to the present study, mixed effect modeling was used by Niemierko et al^10^ when analyzing LET_d_ associations with RAIC in 50 patients treated with PSPT for brain tumors and HNC. In contrast to our findings, the effect of LET_d_ was not found to be significantly correlated with RAIC, neither from the analysis using dose-matched voxels nor by mixed effect logistic regression. Compared with the current study, the heterogeneity in their patient material was higher (ICC of 0.96 vs 0.77), which may be one explanation for the difference in the significance of LET_d_. Further, our patients received treatment with IMPT, which may yield an overall higher LET_d_ compared with PSPT.^30^

Our result showed a more rapid increase in RAIC risks for doses exceeding 60 Gy even for the lowest LET_d_ values, confirming that dose is the main determinant in the development of RAIC.^31^ However, the ability of the model to distinguish between voxels with and without RAIC was improved when including the LET_d_ as predictor. Although our random effect analysis showed large interpatient variation in the LET_d_ effect, it is relevant to consider assessment of the LET distribution in the evaluation of clinical treatment plans, not least because our result showed a clear LET_d_ effect for the majority of the patients. For IMPT, where the LET distributions can be very different for seemingly similar dose distributions,^32^ studies on LET optimized treatment planning have reported promising results, with reduced high LET values in OARs;^11,33,34^ however, this remains an area of active investigation and it is unknown whether this translates into a clinical benefit. Regardless, analyzing clinical outcomes from LET optimized treatment plans may provide useful insight of the importance of LET and increased biologic effectiveness.

Previously, we reported RAIC associations at patient level in a cohort of patients with HNC treated with passive scattering and/or active scanning, finding significant RAIC correlations only for dosimetric variables in the multivariate analysis.^12^ For the current study, where we specifically investigated the spatial relationship between dose, LET_d_ and RAIC, we considered it appropriate to only include patients with RAIC. In future studies, it may be relevant to also include patients without RAIC. To identify potential differences in the LET_d_ distributions between patients with and without RAIC, a matched design with a large patient cohort would be required.

In the present study, we used a mixed effect logistic regression model to investigate the dose and LET_d_ correlations with RAIC. A standard logistic regression model would consider each voxel as an independent observation, ignoring the correlation between the voxels in each patient. Neglecting this clustering structure of the data would affect the parameter estimates and in particular the associated *P* values and CIs. The mixed effect logistic regression model strengthens the result of the current study, as the method controls for nonindependence between voxels.

In addition to the intrinsic shortcomings of a retrospective analysis, the limitations of the present study include the low number of patients in the study cohort and the uncertainties in the dose and LET_d_ values used for modeling due to potentially image registration inaccuracies, proton range uncertainties, and anatomic deformations. Further, the LET_d_ was used as input variable in the models instead of the full LET spectrum. Although it is assumed that the LETs in clinical proton beams are in the range where the RBE increases linearly with LET, and hence are below values where the overkill effect is likely to occur, we cannot rule out that this simplification adds additional uncertainty to the models. Finally, there are uncertainties in the identification of the lesion origin location due to the progressive nature of RAIC. As the MRIs are obtained in a certain time interval during clinical follow-up, RAIC could have been in progression for a period at the earliest available MRI.

In conclusion, using a mixed effect method we found an overall statistically significant dose and LET correlation with RAIC after IMPT for HNC. Despite the large interpatient difference in radiosensitivity, our results suggest that the LET_d_ effect could be of clinical significance for some patients. LET_d_ assessment in clinical treatment plans should therefore be taken into consideration. Future directions include investigating if LET_d_ optimization could reduce observed and predicted RAIC risk without compromising treatment plan and target dose coverage.

## Supplementary Material

Supplementary material

## Figures and Tables

**Table 1:** Patient characteristics (15 patients)

Characteristics	No. (%) or median (range)
Female sex	8 (53.3)
Age	53 (24–71)
Disease site	
Nasopharynx	8 (53.3)
Sinonasal	5 (33.3)
Other	2 (13.3)
T category	
T1–T2	3 (20)
T3–T4	10 (66.7)
Recurrent	2 (13.3)
Unresectable disease	7 (46.6)
Chemotherapy	14 (93.3)
CTV 1 volume (cm^3^)*	110.6 (25.5–340.0)
CTV 2 volume (cm^3^)^†^	194.0 (3.0–484.0)
Number of beams	3 (2–5)
Prescribed dose (Gy[RBE])	70 (60–70)
Number of fractions	33 (30–33)
Fraction dose (Gy[RBE])	2.12 (2.0–2.20)
Time to RAIC (months)	19 (9–33)

*Abbreviations*: CTV = clinical target volume; RAIC = radiation-associated brain image change; RBE = relative biological effectiveness.

* Prescribed dose: 63–70 Gy(RBE).

† Prescribed dose: 57–63 Gy(RBE).Other: orbital and skin.

**Table 2:** Parameter estimates (95% confidence intervals in parenthesis) for the univariate and multivariate models including model fit and performance measures

Variables	Univariate model (LET_d_)	Univariate model (dose)	Multivariate model
LET_d_ (keV/*μ*m)	1.81 (0.79, 2.84)*	-	1.90 (0.56, 3.20)^†^
Dose (Gy)	-	2.72 (1.86, 3.58)*	2.90 (2.00, 3.79)*
LET_d_:dose	-	-	−0.32 (−0.38, −0.26)*
Random effects (SD)			
LET_d_ (keV/*μ*m)	2.09 (1.51, 3.14)	-	2.68 (1.93, 4.93)
Dose (Gy)	-	1.73 (1.24, 2.61)	1.81 (1.30, 2.72)
Model fit and performance			
AIC	101,671.8	76,704.9	70,106.4
Log likelihood	−50,831.9	−38,348.4	−35,046.2
Pseudo R^2^ (fixed effects)	0.37	0.39	0.48
Pseudo R^2^ (total)	0.63	0.83	0.88
Brier score	0.05	0.04	0.04

*Abbreviations*: AIC = Akaike Criteria Information; CI = confidence interval; LET_d_ = dose-averaged linear energy transfer; SD = standard deviation.

* *P* < .001.

† *P* < .01.

LET_d_:dose: interaction term; Pseudo R^2^: Nagelkerke’s.
