# Supplementary material for: Mixed Effect Modeling of Dose and Linear Energy Transfer Correlations With Brain Image Changes After Intensity Modulated Proton Therapy for Skull Base Head and Neck Cancer
Source: Int J Radiat Oncol Biol Phys. Author manuscript; Available in PMC 2022 Feb 18. (PMC8855940; doi:10.1016/j.ijrobp.2021.06.016)
Supplement: Supplementary material [file NIHMS1778281-supplement-Supplementary_material.docx]

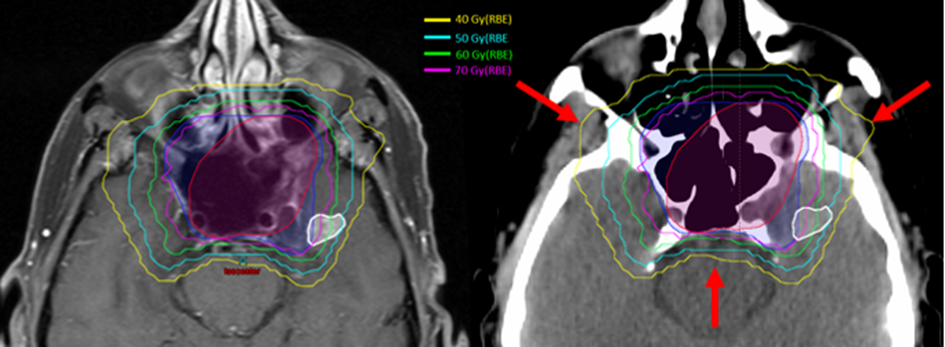


Figure 1: Dose distributions and typical beam directions (red arrows). White contour is contrast enhanced region from MRI (left) and on planning CT (right). Yellow, cyan , green and magenta isodose lines represents 40 GyRBE, 40, 60 and 70 GyRBE, respectively. Red contour is CTV-70 GyRBE and blue contour is CTV-66 GyRBE.


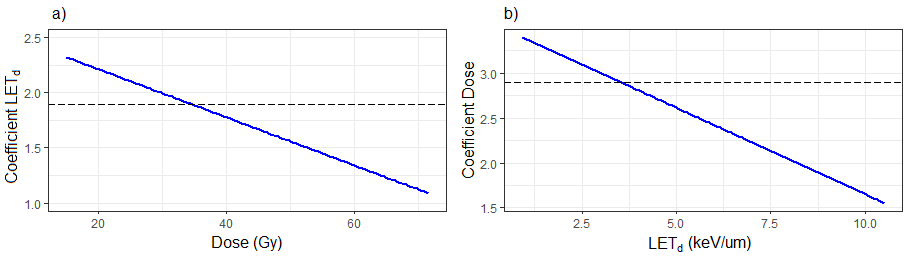


Figure 2: Conditional effects of dose and LET_d_ for the main model. As dose increases, the effect of LET_d_ decreases (a) and vice versa (b). Further, the negative interaction between LET_d_ and dose means that the combined effect of LET_d_ and dose is less than the sum of the individual effects.


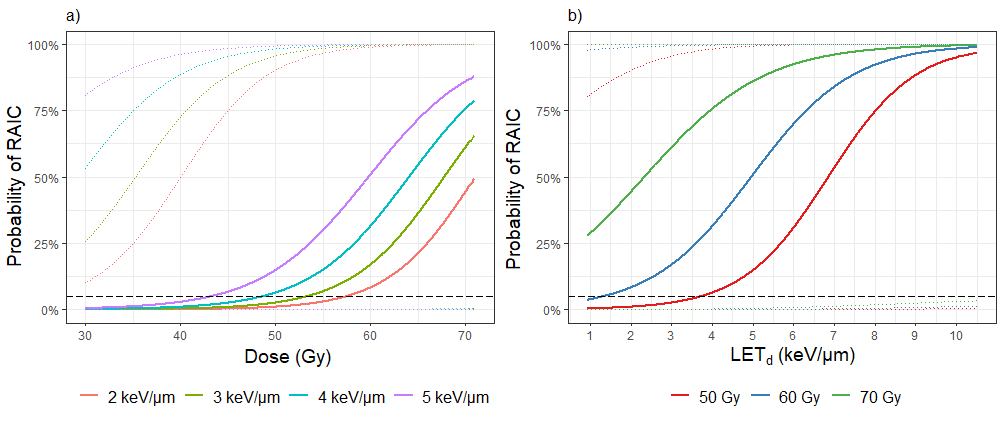


Figure 3: Probability curves (solid lines) including 95% prediction intervals (dotted lines) for four different values of LET_d_ (a) and three different dose levels (b). In both plots the dashed horizontal line corresponds to 5% probability of RAIC.

Table I: Result from subgroup analysis of mixed effect regression modelling for voxel with dose > 40 Gy

|  |  |  |  |  |
| --- | --- | --- | --- | --- |
|  | Univariate | | Multivariate | |
| Parameter | Coefficient | Random effects | Coefficient | Random effects |
| LET_d_ (keV/µm) | 1.17 (0.15, 2.18)* | 1.87 (1.35, 2.82) | 1.46 (0.24, 2.69)** | 2.26 (1.63, 3.40) |
| Dose (Gy) | 1.10 (0.50, 1.70)*** | 1.10 (0.80,1.66) | 1.14 (0.43, 1.85)*** | 1.31 (0.95,1.97) |
| LET_d_:dose |  |  | -0.55 (-0.60,0-0.51)*** |  |
| Random effects: standard deviation, p < 0.05, **p < 0.01, ***p < 0.001. LET_d_: dose: interaction term | | | | |


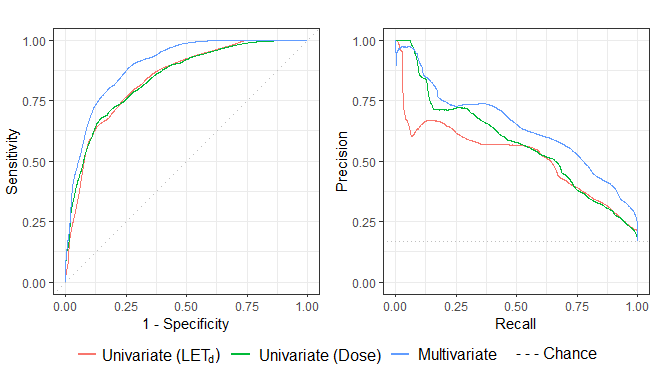


Figure 4: Result from the subgroup analysis. ROC- and PR curves for the univariate and multivariate models displayed at the left and right figure, respectively. The AUC were 0.84 for both the univariate models and 0.90 for the multivariate model. The PR-AUC were 0.52 for univariate model with LET_d_ as predictor, 0.57 for the model with dose as predictor and 0.63 for the multivariate model. Dotted lines represents a no-skill model.
